# Hot-Melt Extrusion as an Effective Technique for Obtaining an Amorphous System of Curcumin and Piperine with Improved Properties Essential for Their Better Biological Activities

**DOI:** 10.3390/molecules28093848

**Published:** 2023-05-01

**Authors:** Kamil Wdowiak, Robert Pietrzak, Ewa Tykarska, Judyta Cielecka-Piontek

**Affiliations:** 1 Department of Pharmacognosy, Faculty of Pharmacy, Poznan University of Medical Sciences, Rokietnicka 3, 60-806 Poznan, Poland; kamil.wdowiak@student.ump.edu.pl; 2 Faculty of Chemistry, Adam Mickiewicz University in Poznań, Uniwersytetu Poznańskiego 8, 61-614 Poznan, Poland; pietrob@amu.edu.pl; 3 Department of Chemical Technology of Drugs, Poznan University of Medical Sciences, Grunwaldzka 6, 60-780 Poznan, Poland; etykarsk@ump.edu.pl

**Keywords:** amorphous systems, curcumin, piperine, hot-melt extrusion, bioaccessibility, delivery system

## Abstract

Poor bioavailability hampers the use of curcumin and piperine as biologically active agents. It can be improved by enhancing the solubility as well as by using bioenhancers to inhibit metabolic transformation processes. Obtaining an amorphous system of curcumin and piperine can lead to the overcoming of these limitations. Hot-melt extrusion successfully produced their amorphous systems, as shown by XRPD and DSC analyses. Additionally, the presence of intermolecular interactions between the components of the systems was investigated using the FT-IR/ATR technique. The systems were able to produce a supersaturation state as well as improve the apparent solubilities of curcumin and piperine by 9496- and 161-fold, respectively. The permeabilities of curcumin in the GIT and BBB PAMPA models increased by 12578- and 3069-fold, respectively, whereas piperine’s were raised by 343- and 164-fold, respectively. Improved solubility had a positive effect on both antioxidant and anti-butyrylcholinesterase activities. The best system suppressed 96.97 ± 1.32% of DPPH radicals, and butyrylcholinesterase activity was inhibited by 98.52 ± 0.87%. In conclusion, amorphization remarkably increased the dissolution rate, apparent solubility, permeability, and biological activities of curcumin and piperine.

## 1. Introduction

Plant materials are rich sources of plant-active substances with great potential to improve human health. They can act as both prophylactic agents and medicines for various disorders. Turmeric (*Curcuma longa* L.) has long been recognized as a natural remedy for many ailments, which is evident when studying ancient Chinese medicine or Ayurvedic practices [1]. The main constituent of turmeric is curcumin; therefore, the health benefits of plant material can be attributed to the presence of this phytochemical. In many studies, the vast biological activity of curcumin was proven, such as its antioxidant, anti-inflammatory, anti-diabetic, anti-cancer, and neuroprotective properties [2,3,4].

Another plant recognized as valuable is the black pepper (*Piper nigrum* L.). The main active substance in this popular spice is an alkaloid compound named piperine. This compound was found to possess a wide range of biological activities such as antioxidant, anti-tumor, anti-inflammatory, antidepressant, immunomodulatory, and neuroprotective effects [5,6,7]. Piperine is also said to be a bioenhancer, which means that it increases the bioavailability of co-administered compounds. This observation was supported by Bi Xiaoli et al., who reported that piperine increased the bioavailability of silybin when supplemented simultaneously [8].

Unfortunately, the therapeutic potential of both compounds cannot be fully utilized since they are poorly soluble compounds [9,10], which determines their inability to generate sufficient blood concentration and enough active substance at the site of the molecular target. Apart from poor solubility, which affects poor bioaccessibility, an obstacle in curcumin’s bioavailability is also an extensive first-pass effect. Curcumin is primarily converted to its glucuronide and sulfate metabolites [11]. Furthermore, it is a substrate of glycoprotein-P, which results in curcumin being pumped out of the cells [12].

To address the issue of low bioavailability, systems for the simultaneous delivery of curcumin and piperine were developed. For instance, Kazi et al. produced the self- nanoemulsifying system to co-deliver curcumin and piperine [13]. The systems presented an amorphous character, enhanced the solubilization capacity of curcumin and piperine, and improved their release profiles. Chen et al. prepared zein–carrageenan core–shell nanoparticles loaded with curcumin and piperine, which increased the thermal and light stability of the compounds as well as provided controlled release [14]. Furthermore, nanoparticles enhanced the antioxidant activity of plant-origin active substances. Tang et al. obtained solid lipid nanoparticles with tocopheryl polyethylene glycol succinate and Brij 78 to co-deliver curcumin and piperine [15]. This system enhanced cytotoxic activity against drug-resistant cancer cells as well as allowed efficient delivery of active compounds into the cells. Abolhassani et al. obtained self-assembling serum albumin nanoparticles co-loaded with curcumin and piperine [16]. The system demonstrated high anti-cancer potential against breast cancer cells with the ability to suppress the multidrug resistance effect. Javed et al. fabricated lignin-g-p (NIPAM-co-DMAEMA) gold nanogels loaded with curcumin and piperine [17]. The systems were cytotoxic against glioblastoma multiforme and triggered caspase-3-mediated apoptosis.

One of the effective methods for increasing the solubility of active substances is amorphization. The solubility and dissolution rate of compounds are markedly increased when the crystalline structure is broken and an amorphous state is attained [18,19]. Out of many available techniques that enable the fabrication of amorphous systems, hot-melt extrusion seems to be one of the best choices. It offers several benefits, such as a solvent-free approach, the relatively low-cost and continuous character of the process, easy scale-up to the industrial level, and an improvement in solubility since it promotes the formation of amorphous systems [20,21].

Both of the tested compounds, curcumin and piperine, are characterized by poor solubility; therefore, it is crucial to improve their bioaccessibility to fully benefit from their biological potential and use their combination as a prophylactic and treatment agent. This work set out to produce amorphous curcumin–piperine dispersions with enhanced solubility for both substances. The research was divided into three stages: (1) amorphous system preparation through hot-melt extrusion; (2) amorphous system identification and characterization of the physicochemical properties of its components, such as dissolution rate, solubility, and permeability; and (3) assessment of biological activity, including antioxidant and neuroprotective potential.

## 2. Results and Discussion

Curcumin and piperine possess promising biological properties, but due to their low solubility and associated poor bioavailability, their utility in the treatment of many disorders is hampered. Low blood concentrations result in a lack of noticeable pharmacological effects. The purpose of this research is to create amorphous systems to deal with these problems.

So far, there have been several attempts to connect curcumin and piperine in an amorphous system to improve bioaccessibility and overall bioavailability. Wang et al. used a melting and quench-cooling approach to fabricate an excipient-free co-amorphous system [22]. Prepared systems were stable in the aging test, and there was an improvement in the apparent solubility of tested compounds. In the Caco-2 study, authors reported enhanced permeability as well as inhibition of the glucuronidation of curcumin. However, this research involved an amorphization technique that is hardly applicable on a larger scale. Next, Althobaiti et al. developed curcumin–piperine amorphous dispersions via hot-melt extrusion using Soluplus as a carrier [23]. They reported improvements in curcumin releases of up to 9-fold. Furthermore, no recrystallization was reported after 3 months. This study strongly focused on the characterization of the systems, omitting the impact of amorphous dispersion on permeability and biological potential. In the aforementioned papers, the authors firmly supported the idea of curcumin–piperine amorphous systems, suggesting some approaches to their production. However, through our work, we would like to offer a different line of action and further check the impact of the modification on permeability as well as biological potential by using simple and accessible in vitro methods.

In our research, the systems were prepared via an environmentally friendly approach using hot-melt extrusion. We used Kollidon VA64 (PVP VA64) as a polymeric carrier acting as an inhibitor of crystallization. The systems were prepared at various mass ratios.

For the identification of amorphous systems, techniques such as XRPD, DSC, and FT-IR were used.

**Figure 1:**
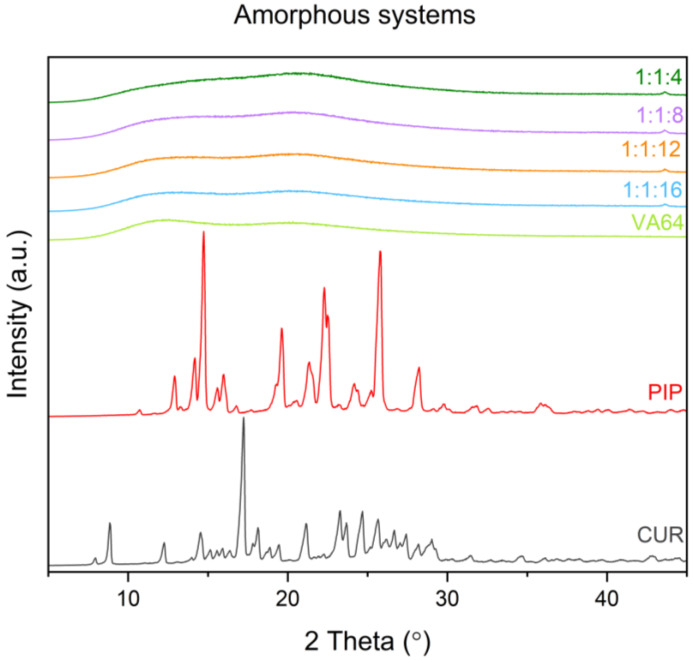
Diffractograms of raw compounds and amorphous systems. Amorphous systems were named as a mass ratio of individual components, Cur:Pip:VA 64 (Cur: curcumin, Pip: piperine, and VA 64: PVP VA 64).

XRPD study is a first-line test to confirm the amorphization of the material. In Figure 1, the diffractograms of raw compounds and amorphous systems are presented. Raw curcumin showed well-defined sharp peaks at 2Theta angles 7.94°, 8.85°, 12.25°, 14.55°, 15.13°, 15.53°, 15.89°, 16.36°, 17.24°, 18.14°, 18.87°, 19.45°, 21.16°, 23.27°, 24.67°, 24.68°, 25.63°, 27.40°, 28.17°, and 29.00°, while raw piperine at 10.69°, 12.91°, 14.15°, 14.73°, 15.60°, 15.97°, 16.76°, 19.64°, 21.34°, 22.29°, 24.19°, 25.81°, 28.21°, and 29.77°. In XRPD patterns of obtained systems, as well as the polymeric carrier PVP VA64, no peaks could be detected, confirming the amorphous character of these materials. In the Supplementary Materials, the XRPD patterns of the systems’ physical mixtures are given (Figure S1).

**Figure 2:**
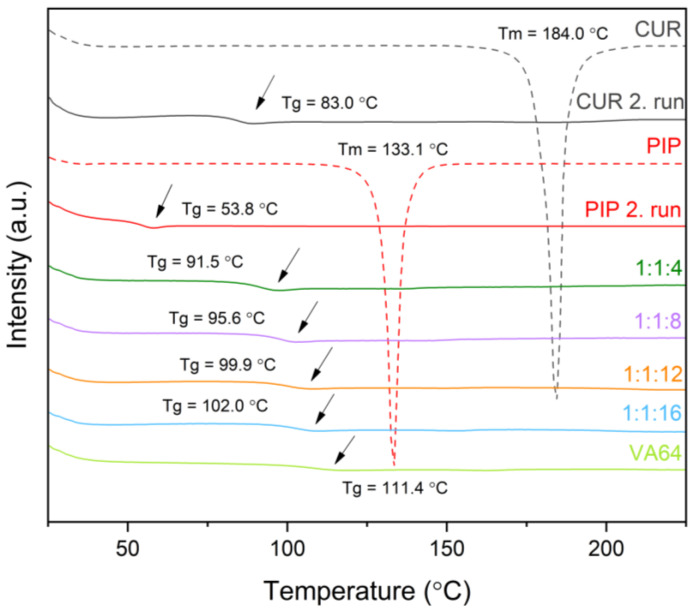
Thermograms of raw compounds and amorphous systems. Amorphous systems were named as a mass ratio of individual components, Cur:Pip:VA 64 (Cur: curcumin, Pip: piperine, and VA 64: PVP VA 64). Arrows point the Tg values.

The DSC study is a basic method to characterize amorphous systems. As can be seen in Figure 2, raw curcumin showed a sharp endothermic peak at 184.0 °C and raw piperine at 133.1 °C. These thermal events correspond to the melting points of individual compounds. The second heating run in DSC was applied to assess the glass transition temperatures of compounds. The Tg value of curcumin turned out to be 83.0 °C, while the Tg of piperine was 53.8 °C. To obtain the thermograms of amorphous systems, first, the samples were heated up to 80 °C and maintained at this temperature for 8 min to get rid of water absorbed by the polymer, then cooled down to 25 °C and heated again to 225 °C. The DSC patterns of amorphous systems revealed a single Tg, indicating the mixing of the components at the molecular level. Moreover, the lack of endothermic peaks that could correspond to melting points is a piece of evidence supporting the statement of systems’ amorphousness, since no parts of crystalline materials are detected. One can notice that the Tg value of the systems increases with the increasing amount of polymer in the systems, meaning that molecular mobility is more hampered when more of the polymeric carrier is introduced into the system. This behavior is caused by the antiplasticization effect exerted by the polymer [24] and might affect physical stability. It is expected that the 1:1:16 system will show the best physical stability and prevent crystallization because it has the highest Tg value (Tg = 102.0 °C).

After amorphization confirmation, we proceeded to check if there were any molecular interactions formed between the system’s components.

**Figure 3:**
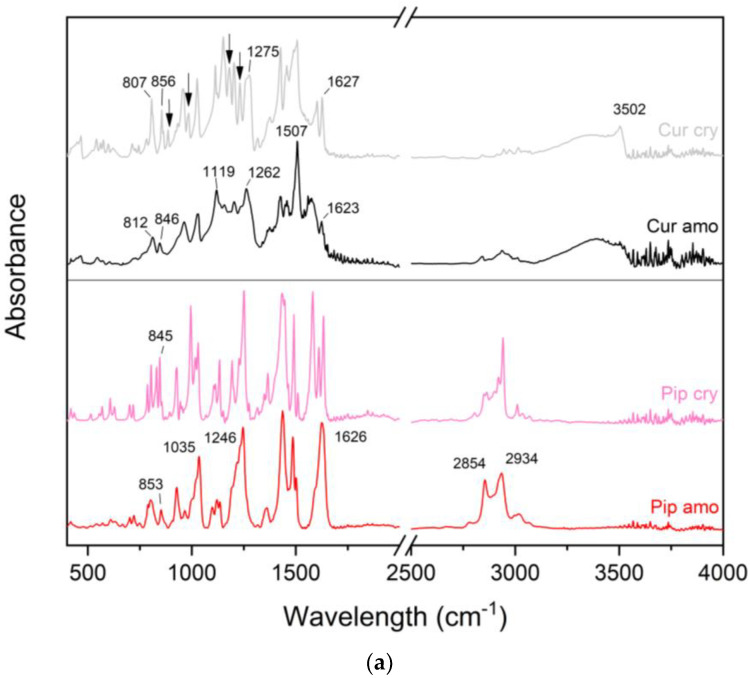

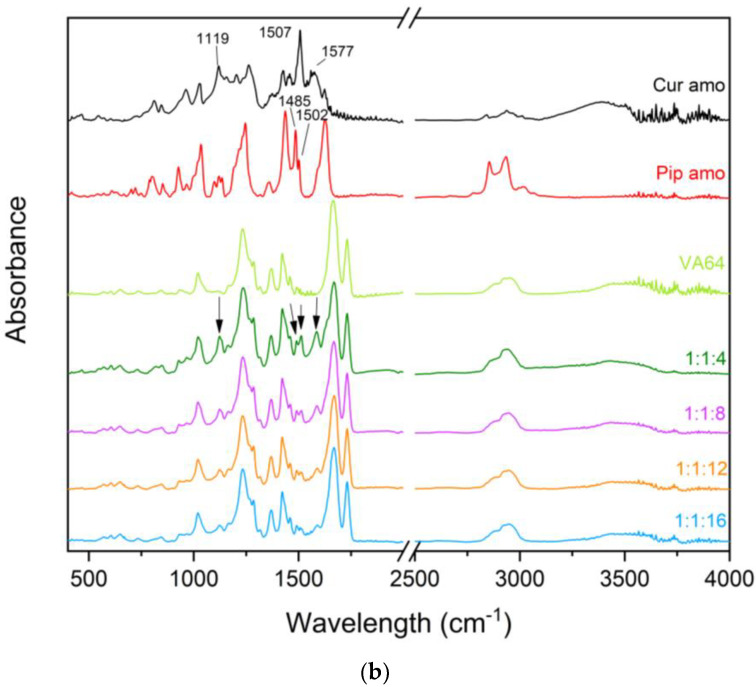
FTIR-ATR spectra of raw curcumin and piperine in crystalline and amorphous forms (**a**), as well as amorphous raw compounds, Kollidon VA 64, and amorphous systems (**b**). Amorphous systems were named as a mass ratio of individual components, Cur:Pip:VA 64 (Cur: curcumin, Pip: piperine, and VA 64: PVP VA 64). Black arrows indicate changes.

When we compare raw crystalline compounds and their amorphous forms, the FT-IR/ATR spectra show noticeable differences (Figure 3a). The FT-IR/ATR spectra of crystalline curcumin show a sharp peak at 3502 cm^−1^, which fades into an amorphous form, and the disappearance of small bands at 3071 and 3015 cm^−1^ is also seen. The bands at 2971 and 2945 cm^−1^ fused into one broad band with a maximum at 2937 cm^−1^ in amorphous form. The peak at 1627 cm^−1^ decreased in intensity and slightly shifted to 1623 cm^−1^. The peak at 1601 cm^−1^ flattened out. In contrast, the peak at 1507 cm^−1^ sharpened considerably, and the one at 1426 cm^−1^ flattened out. One can note the shift of the peak from the maximum at 1275 to 1262 cm^−1^ and the disappearance of the 1231 and 1180 cm^−1^ peaks. Then, 1152 and 1113 cm^−1^ peaks reduced in intensity and shifted to 1157 and 1119 cm^−1^, respectively. The peaks at 985 and 886 cm^−1^ disappeared, and the one at 959 cm^−1^ widened its base. Finally, the peaks at 856 and 807 cm^−1^ flattened out, and their maxima shifted to 846 and 812 cm^−1^, respectively. In the 800–400 cm^−1^ range, in particular, a significant reduction in intensity can be observed. In the case of piperine, the broad peak with several maxima at 2942, 2919, 2863, and 2849 cm^−1^ fused into a single broad peak with two absorption maxima at 2934 and 2854 cm^−1^. The peak with three separate maxima at 1634, 1609, and 1582 cm^−1^ merged, creating a peak with a maximum at 1626 cm^−1^. The peaks at 1512, 1490, and 1433 cm^−1^ had their bases widened, leading them to combine yet retain their maxima in the case of amorphous form. Peaks at 1249, 1227, and 1192 cm^−1^ converged into a single peak with a maximum at 1246 cm^−1^. The same was observed for the peaks at 1029, 1018, and 994 cm^−1^, which fused into one with the maximum at 1035 cm^−1^. The 845 cm^−1^ peak shifted to 853 cm^−1^, the 829 cm^−1^ peak vanished, and the 786 cm^−1^ peak fused with the 802 cm^−1^ peak. Reduced intensity and broadening of peak bases can be noted in the 750–400 cm^−1^ range.

In order to confirm the occurrence of interactions between the components of the systems, the spectra of the amorphous pure compounds as well as the carrier were compared with the spectra of the prepared systems (Figure 3b). As for the FT-IR/ATR spectra of the amorphous systems, it can be seen that a well-pronounced peak appears at 1586 cm^−1^, which may be a shifted 1577 cm^−1^ peak of curcumin. Also visible is a broad-based peak with two maxima at 1514 and 1489 cm^−1^, which may be a shifted piperine peak that also has two maxima at 1502 and 1485 cm^−1^, although the system’s peak at 1514 cm^−1^ can also be assigned to a shifted curcumin peak at 1507 cm^−1^. On the other hand, the peak at 1123 cm^−1^ may be a shifted 1119 cm^−1^ peak of curcumin. Individual bands can be attributed to bands of individual components. The observed changes suggest the formation of interactions in the obtained amorphous systems. The positions of the most important peaks of raw curcumin, piperine, and Kollidon VA64 are given in the Supplementary Materials (Table S1).

Physical stability needs to be considered when formulating amorphous systems. To fully benefit from the amorphization process in medical products, the amorphous state needs to be maintained after production, on a pharmacy shelf. To initially assess the physical stability, the systems were stored at two different environmental conditions: ambient conditions (shelf conditions) as well as at an increased temperature (50 °C) without humidity control. The systems were stable in both storage conditions after 6 months, as the XRPD study revealed. The diffractograms regarding physical stability are given in the Supplementary Materials (Figure S2).

There is a general rule concerning the physical stability of amorphous systems. It is said that amorphous systems are expected to be physically stable if the glass transition temperature of the system is 50 °C above the storage temperature [25]. Interestingly, some of the obtained systems present a Tg value less than 100 °C, meaning that at elevated temperatures up to 50 °C, they should tend to crystallize, and yet after 6 months in those conditions, they remained amorphous. These observations might be a piece of evidence that, apart from the restriction of molecular mobility provided by the polymeric carrier, a different mechanism is responsible for the stability of the amorphous state, such as intermolecular interactions [26,27]. However, it could be the case that the time applied for physical stability studies is too short to determine stability over a long period of time, extending to a year. All in all, the physical stability presented by amorphous systems is a good indicator of system potential, supporting the idea of further development of these formulations.

After confirming the nature of the prepared systems, we evaluated the effects of amorphous dispersions on the parameters important from a pharmaceutical point of view. Tests were conducted to characterize the physicochemical features, such as dissolution rate, solubility, and permeability. The biological potential of the systems was then examined to see if the amorphization had a favorable effect.

**Figure 4:**
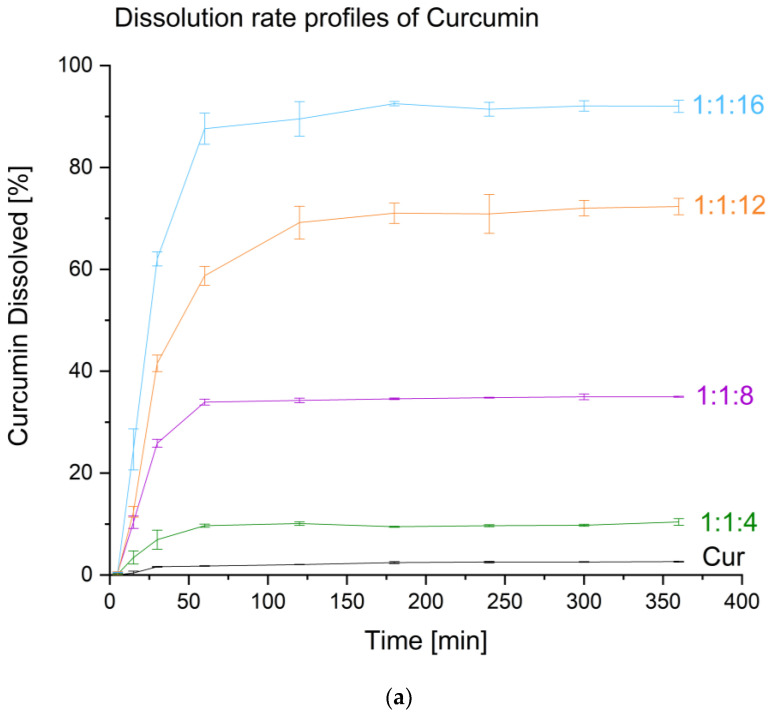

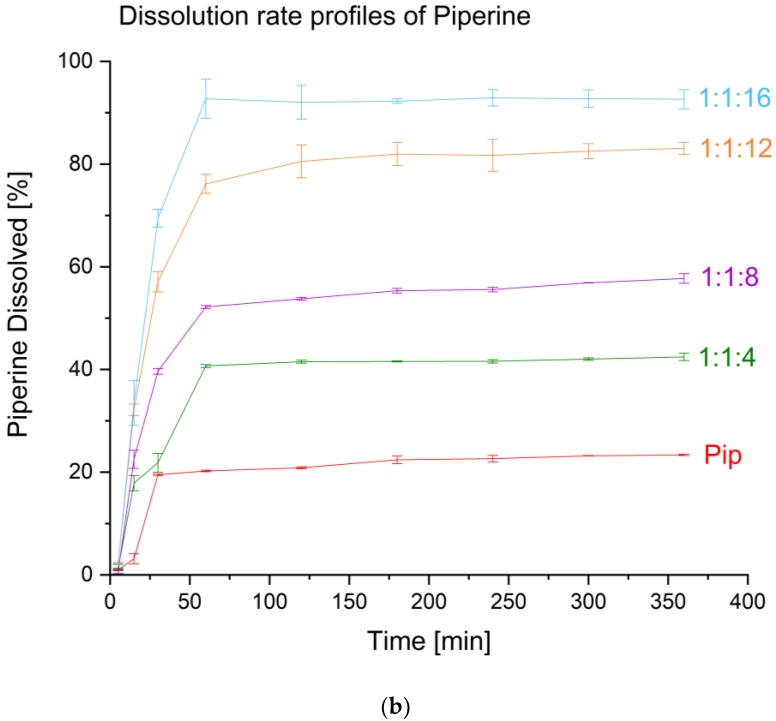
Dissolution rate profiles for amorphous systems of curcumin (**a**) and piperine (**b**). Amorphous systems were named as a mass ratio of individual components, Cur:Pip:VA 64 (Cur: curcumin, Pip: piperine, and VA 64: PVP VA 64).

One of the beneficial features of amorphous dispersions is that they enhance the dissolution rate profile. The obtained amorphous dispersions made sure that a supersaturated state was obtained because the dissolution profiles for both substances indicated an increase in the level of apparent solubility compared to raw compounds. Both times, the amorphous systems reached a plateau after roughly an hour and held it for the full six hours of the test. Raw curcumin dissolved to a level of 2.54 ± 0.15% after one hour, whereas raw piperine reached 22.63 ± 0.67% of the dose supplied to the dissolution medium (Figure 4a,b). Regarding the amorphous systems, the curcumin–piperine–VA64 system in a mass ratio offered the greatest improvement in apparent solubility (1:1:16). It allowed the dissolution of 91.42 ± 1.36% of curcumin and 92.74 ± 1.60% of piperine. The 1:1:16 system provided the best improvement in the dissolution rate profile for both compounds compared to other ratios. One can explain this by the fact that there is more of the polymer in this system, which can stabilize the amorphous state in the solution and also provide better wettability of the system itself, which led to an almost complete release of the compounds from the polymer matrix as well as the maintenance of the supersaturated state in the solution.

Produced amorphous systems generated and enabled the maintenance of the supersaturated state. This fact provides a piece of evidence that Kollidon VA64 prevented the model compounds from crystallizing. In the dissolution rate study, curcumin and piperine both showed an increase in apparent solubility to the extent of 36-fold and 4-fold, respectively. The amorphous state provides improved permeability, which translates into higher bioavailability, assuming that it leads to the generation of a supersaturation state, preferably maintained for a time allowing complete absorption of the administered dose. Otherwise, the amorphous formulation will not show its full bioavailability-enhancing potential. It is well known that an amorphous state and the accompanying creation of supersaturation are responsible for enhanced bioavailability. Some studies provide pieces of evidence supporting this observation. Kwon et al. prepared amorphous systems of atorvastatin via spray-drying that increased their apparent solubility [28]. Furthermore, after oral administration, systems demonstrated higher AUC and Cmax than the raw drug. Qin et al. then used a solvent evaporation method to create amorphous dispersions of nintedanib [29]. Their work showed that the addition of a polymer to amorphous systems delays or inhibits precipitation and crystallization, thus contributing to the remaining supersaturation. Furthermore, the authors demonstrated that amorphous dispersion with the polymer prolonged the absorption period and provided a sustained plasma profile of the drug, allowing it to achieve a higher AUC value than the raw drug.

It is worth noting that the systems ensured the maintenance of the supersaturation state for up to 6 h, which is the approximate time that intestinal content can remain in the duodenum, the major site of absorption in the body [30]. Using an amorphous dispersion does not immediately imply that the supersaturation lasts over time. The behavior of the active ingredient depends on the carrier and the degree to which crystallization is prevented. It can happen that the parachute effect is not well-pronounced, and thus the concentration of the drug starts to fall after a relatively short time from the observation of the spring effect. The study of Butreddy et al. supports this statement since in their research, the binary system of nifedipine-HPMCAS-LG showed a high supersaturation concentration, but it dropped in a short period of time and was not maintained up to 6 h of study [31].

The supersaturation generated by amorphous systems can directly translate into better absorption of active substances since only free, dissolved molecules can penetrate biological barriers. Some studies suggest that supersaturation contributes to increased drug levels in the blood [32,33,34]. Accordingly, the resulting amorphous dispersion may also allow for increased absorption of curcumin and piperine. In addition, since the apparent solubility of the compounds was markedly increased, i.e., 9496-fold for curcumin and 161-fold for piperine, the bioavailability improvement potential is tremendous (Table 1). Raw piperine has better crystalline solubility than curcumin. In contrast, in systems, curcumin has higher solubility; this may be due to the fact that Kollidon VA64 better stabilizes the amorphous state of curcumin than piperine. The reason could be the polymer’s preferential interactions with curcumin.

After determining the remarkable effect of the obtained amorphous systems on the dissolution rate and solubility, we proceeded to check whether they affect the permeability of compounds. PAMPA models were carried out to simulate passive diffusion through intestinal walls (GIT model) and across the blood–brain barrier (BBB model) in order to ascertain whether the prepared systems resulted in improved permeability. An apparent permeability coefficient factor was calculated to see if the model compounds themselves have a tendency to passively go through the aforementioned biological barriers. It was discovered that the values for curcumin and piperine for PAMPA GIT were 1.63 × 10^−6^ ± 6.05 × 10^−8^ cm/s and 8.18 × 10^−6^ ± 5.64 × 10^−6^ cm/s, respectively. This leads to the conclusion that the investigated compounds exhibit good gut penetration. In PAMPA BBB, the estimated permeability factors for curcumin and piperine were 2.63 × 10^−6^ ± 8.43 × 10^−7^ cm/s and 5.77 × 10^−5^ ± 2.96 × 10^−6^ cm/s, respectively. This implies that these compounds will be able to pass through the blood–brain barrier as well. The concentrations reached in the acceptor part were placed side by side to compare the permeability of the raw compounds and those in the amorphous systems (Table 2). Here, one can clearly observe that in both PAMPA models, permeability improved. In the PAMPA GIT model and the PAMPA BBB model, the concentration of raw curcumin was found to be as low as 3.34 × 10^−6^ ± 1.94 × 10^−6^ mg/mL and 1.86 × 10^−5^ ± 2.48 × 10^−6^ mg/mL, respectively. The best system improved permeability, allowing it to reach 4.20 × 10^−2^ ± 2.21 × 10^−3^ mg/mL in the GIT model and 5.70 × 10^−2^ ± 1.40 × 10^−3^ mg/mL in the BBB model, indicating that these parameters increased by 12578-fold and 3069-fold, respectively. For piperine, similar findings were recorded. The concentration in the acceptor part increased 343-fold in the GIT model and 164-fold in the BBB model, respectively, from 5.86 × 10^−4^ ± 1.35 × 10^−4^ mg/mL to 2.01 × 10^−1^ ± 7.77 × 10^−3^ mg/mL and from 2.10 × 10^−3^ ± 8.86 × 10^−5^ mg/mL to 3.45 × 10^−1^ ± 2.07 × 10^−2^ mg/mL. Overall, the finding from permeability experiments supports what was discovered through the solubility study. The increase in solubility supported the passive diffusion of curcumin and piperine, resulting in higher concentrations of these compounds in the acceptor part, as PAMPA models stated. The poor solubility of curcumin and piperine restricts their bioavailability because they both easily pass through biological barriers.

One should keep in mind that the PAMPA study is able to assess the diffusion potential; however, in the human body, there are also other mechanisms affecting the overall permeability and, as a result, the blood concentrations of plant-origin compounds. In the case of curcumin, it is worth mentioning that curcumin bioavailability is also affected by active efflux, further hampering absorption. Efflux proteins such as glycoprotein-P, MRP2, and BCRP contribute to the outflow of curcumin back to the intestinal lumen, thus reducing the level of absorption of the compound [35]. Interestingly, curcumin is able to inhibit efflux active transporters to some extent, such as BCRP and MRP2, not only promoting the absorption of itself but also co-administered compounds [36,37].

Despite having a low oral bioavailability, curcumin can pass biological barriers such as the blood–brain barrier, because of its lipophilicity. Therefore, it can promote neuroprotection by reaching the brain. However, it should be noted that curcumin enters the brain only under the condition that it is not glucuronidated [38]. In this case, the suppression of metabolism by piperine might decrease the level of glucuronidated curcumin in the blood, meaning that the neuroprotective activity would be promoted.

Bearing in mind the significant improvement in the solubility of curcumin and piperine and the impact on the penetration through membranes simulating the GIT and BBB barriers, the authors decided to evaluate the activity of both compounds on in vitro models connected to neuroprotective potential. Therefore, the ability to improve antioxidant properties (with regard to DPPH radical) and decrease butyrylcholinesterase (BChE) activity was examined. It is clear that the process of amorphization and the resulting increase in solubility had a positive effect on biological activity. The Cur:Pip:VA 64 1:1:16 system, which reduced the DPPH radical at 96.97 ± 1.32% and suppressed BChE activity at 98.52 ± 0.87%, displayed the highest activity. Interestingly, 1:1:12 and 1:1:16 (Cur:Pip:VA64) systems showed statistically similar DPPH inhibition ability. In contrast, a physical mixture of raw compounds in a 1:1 mass ratio produced 10.69 ± 0.92% and 1.24 ± 0.27% inhibition in the DPPH and BChE tests, respectively. The results of biological activity assays are given in Table 3. One can notice that in the antioxidant activity test using the DPPH radical, the 1:1:12 and 1:1:16 systems show the same activity. These observations may be due to the sensitivity of the test itself, as both systems showed maximum activity; therefore, neither can be selected as a better one, although in other studies, the 1:1:16 system is clearly superior.

The combination of curcumin and piperine exhibits neuroprotective synergism, as demonstrated in several studies. Singh et al., in a rat model of Huntington’s disease, showed that the combination of curcumin and piperine improved motor function more effectively than curcumin alone, increased antioxidant activity by increasing glutathione levels, had anti-inflammatory effects by reducing TNF-α and IL-1b levels, and improved neurotransmitter levels in the striatum [39]. In turn, Bishnoi et al., on a haloperidol-associated neurotoxicity model, showed that simultaneous administration of curcumin and piperine leads to increased levels of neurotransmitters, reduced oxidative stress, and decreased production of inflammatory response mediators [40]. In turn, Erfen et al. reported improved cell survival and a decrease in apoptosis and in the number of cytokines IL-6 and TGF-β in aluminum-treated astrocyte cells, which the authors attributed, among other things, to piperine increasing the effectiveness of curcumin [41]. Abdul Manap et al. showed a synergistic effect of curcumin and piperine in an Alzheimer’s disease model, where a combination of these plant-origin active substances strengthened the inhibitory effect on AchE and led to an increase in cell viability due to protection from B-amyloid-induced neurotoxicity [42]. Furthermore, combining curcumin and piperine demonstrated an anti-amyloidogenic effect via the inhibition of aggregation and disaggregation of β-amyloid proteins as well as the reversal of amyloid-induced oxidative stress. When discussing the development and progress of neurodegenerative diseases such as Alzheimer’s disease, as well as potential molecular targets during drug design, oxidative stress and esterase enzymes are critical factors [43,44]. In this study, the biological potential of the obtained systems was tested in antioxidant as well as butyrylcholinesterase assays. Both curcumin and piperine are reported to have the capability of inhibiting BChE [45,46]. Curcumin and piperine were developed into amorphous ternary systems, which greatly increased antioxidant activity up to 96.97 ± 1.32% and BChE inhibitory activity to the extent of 98.52 ± 0.87% for 1:1:16 (Cur:Pip:VA64) system. Since only free molecules might interact with the DPPH radical or BChE enzyme and so reduce their activity, these effects are most likely attributable to the fact that solubility has improved. Similar statements were made by Yen et al.; in their study, they developed curcumin nanoparticles that markedly improved water solubility and, as a result, antioxidant and anticancer activities [47].

## 3. Materials and Methods

### 3.1. Materials

All materials including piperine (purity > 95%, FG) were supplied by Sigma-Aldrich (Sigma-Aldrich, St. Louis, MO, USA), except for curcumin (purity > 95%, purchased from Xi’an Tian Guangyuan Biotech Co., Ltd., Xi’an, Shaaxi Province, China), vinylpyrrolidone-vinyl acetate copolymer (Kollidon^®^VA64, PVP/VA 64, BASF, Ludwigshafen am Rhein, Germany), dimethyl sulfoxide (DMSO, POCH, Gliwice, Poland), sodium hydroxide (Avantor Performance Materials Poland S.A., Gliwice, Poland), acetic acid 98–100% (POCH, Gliwice, Poland), sodium dihydrogen phosphate (PanReac AppliChem ITW Reagents, Darmstadt, Germany), and acetonitrile of an HPLC grade (J. T. Baker, Center Valley, PA, USA). High-quality pure water was prepared using a Direct-Q 3 UV purification system (Millipore, Molsheim, France; model Exil SA 67120). Prisma HT, GIT/BBB lipid solution, and acceptor sink buffer were supplied by Pion Inc. (Forest Row, East Sussex, U.K.).

### 3.2. Preparation of the Systems

The hot-melt extrusion process was performed in a HAAKE MiniCTW micro-conical twin screw extruder (Thermo Scientific, Karlsruhe, Germany). First, individual components of systems were mixed with a mortar and pestle in a defined ratio of components, which were 1:1:4, 1:1:8, 1:1:12, and 1:1:16 (curcumin:piperine:Kollidon^®^VA64). Then, physical mixtures were fed manually into the hopper of the extruder at a barrel temperature of 150 °C and a screw speed of 100 rpm. The prepared extrudates were powdered by ball milling using a Retsch Mixer Mill MM 400 (Retsch GmbH, Haan, Germany). Manually crushed extrudates were placed in a 25 mL stainless steel jar with 3 balls ø 10 mm. The milling process lasted 5 min. The powder obtained was used for further studies.

### 3.3. Solid-State IDENTIFICATION

#### 3.3.1. X-ray Powder Diffraction (XRPD)

The crystallographic structure of the samples was analyzed by an X-ray powder diffraction at ambient temperature using a Bruker AXS D2 Phaser diffractometer (Bruker, Germany) with CuKα radiation (1.54060 Å). The tube voltage and current were 30 kV and 10 mA, respectively. The samples were scanned from 5° to 40° 2 Theta with a step size of 0.02° and a counting rate of 2 s·step^−1^. The analysis of the acquired data was performed using Origin 2021b software (OriginLab Corporation, Northampton, MA, USA).

#### 3.3.2. Differential Scanning Calorimetry (DSC)

Thermal analysis was performed using a DSC 214 Polyma differential scanning calorimeter (Netzsch, Selb, Germany). Samples of about 5–8 mg were placed in crimped aluminum pans with a small hole in the lid. First, the samples were heated up to 80 °C and kept at this temperature for 8 min to remove water from the samples; then, they were cooled down to 25 °C and heated again to 225 °C. To measure the glass transition value of raw compounds, they were heated up to 225 °C, and then cooled down and heated again to 225 °C. The measurements were performed at a constant heating rate of 20 °C/min under a nitrogen atmosphere with a flow rate of 30 mL/min. The glass transition value was taken as a midpoint between on-set and end-point temperatures.

#### 3.3.3. Fourier Transform Infrared Spectroscopy with Attenuated Total Reflectance (FTIR-ATR)

The FTIR-ATR spectra were measured between 400 cm^−1^ and 4000 cm^−1^, with the resolution set to 1 cm^−1^, with a Shimadzu IRTracer-100 spectrometer (Shimadzu, Kyoto, Japan) equipped with a QATR-10 single bounce, a diamond extended range, and LabSolutions IR software (Warsaw, Poland). Amorphous forms of raw compounds were prepared by DSC by heating them to 225 °C and maintaining that temperature for 5 min.

#### 3.3.4. Physical Stability

The physical stability was monitored for up to 6 months in dry conditions and different temperature conditions: (1) temperature of 25 °C and (2) temperature of 50 °C. After 1, 2, 3, and 6 months, the samples were taken out and checked for the presence of crystallinity by XRPD study.

### 3.4. Physicochemical Characterization

#### 3.4.1. HPLC Conditions

Concentrations of curcumin and piperine during solubility, dissolution rate, and permeability studies were measured by high-performance liquid chromatography with the DAD detector (HPLC-DAD). In this study, a Shimadzu Nexera system (Shimadzu Corp., Kyoto, Japan) was used, equipped with an SCL-40 system controller, a DGU-403 degassing unit, a LC-40B XR solvent delivery module, a SIL-40C autosampler, a CTO-40C column oven, and a SPD-M40 photodiode array detector. For the stationary phase, a Dr. Maisch ReproSil-Pur Basic-C18 100 Å column with 5 µm particle size and 250 × 4.60 mm (Dr. Maisch, Ammerbuch-Entringen, Germany) was used. The mobile phase was acetonitrile:0.1% acetic acid (85:15 *v*/*v*). The mobile phase was vacuum-filtered through a 0.45 µm nylon filter (Phenomenex, Torrance, CA, USA). The experimental conditions were as follows: 0.5 mL/min flow rate, wavelengths of 420 nm for curcumin and 340 nm for piperine, and a column temperature of 30 °C. The injection volume differed depending on the assay. For the solubility study, it was 1 µL, whereas for the dissolution rate and permeability assays, it was 10 µL. The method’s duration was 12 min. The retention times were 7.375 min for curcumin and 8.470 min for piperine. Chromatograms (Figure S3a,b) and validation parameters (Table S2) are given in the Supplementary Materials.

#### 3.4.2. Media for Dissolution and Solubility Studies

Phosphate buffer at pH 6.8 was prepared according to the following description: in a 1000 mL volumetric flask, we placed 250 mL of 0.2 N potassium dihydrogen phosphate solution, then added 112 mL of 0.2 N sodium hydroxide solution, and filled the mixture up to 1000 mL with distilled water. High-quality pure water was prepared using a Direct-Q 3 UV purification system (Millipore, Molsheim, France, model Exil SA 67120).

#### 3.4.3. Dissolution Studies

The dissolution study was performed on the paddle apparatus (Agilent 708-DS dissolution apparatus, Santa Clara, CA, USA). The amount of the compound and systems corresponding to 7.0 mg of each plant-origin active ingredient was added to the gelatin capsule, placed in a spring used as a sinker, and then added to the dissolution medium. The vessels were filled with 500 mL of phosphate buffer, pH 6.8; the temperature was maintained at 37 °C, and the paddles were set at a stirring speed of 50 rotations per minute. The 2.0 mL samples were withdrawn at predetermined time points (5 min, 15 min, 30 min, 1 h, 2 h, 3 h, 4 h, 5 h, and 6 h) with the replacement of equal volumes of temperature-equilibrated media.

#### 3.4.4. Apparent Solubility Studies

An excess amount of raw compounds and systems (corresponding to 1.5 mg of each substance) was placed in a 10 mL glass tube; then, 2.0 mL of phosphate buffer (pH 6.8) was added and left at room temperature for 3 h. The obtained solutions were diluted 1:10 with water, filtered through a 0.2 μm PTFE membrane filter (Sigma-Aldrich, St. Louis, MO, USA), and analyzed using the HPLC method.

#### 3.4.5. Permeability Studies

In vitro gastrointestinal (GIT) and blood–brain barrier (BBB) permeability were studied using the PAMPA (Parallel Artificial Membrane Permeability Assay) models. The sandwich consists of two 96-well microfilter plates. The PAMPA systems contain two chambers: the donor chamber at the bottom and the acceptor chamber at the top. The chambers are separated by a 120 μm thick PVDF membrane coated with a 20% (*w*/*v*) dodecane solution of a lecithin mixture (Pion, Inc., Forest Row, East Sussex, U.K.). The donor and acceptor solutions were provided by the manufacturer. The donor solution was diluted according to manufacturer recommendations and adjusted to pH ≈ 6.8 for GIT application and to pH ≈ 7.4 for BBB application using 0.5 M NaOH. The plates were combined and then incubated for 3 h for both models in a humidity-saturated atmosphere with the temperature set at 37 °C. To assess the apparent permeability coefficient factor (*Papp*), 5.0 mg of raw compounds were dissolved in 1.0 mL of DMSO. Then, we followed the manufacturer’s guidelines for further performance in the assay. The *Papp* factor was calculated according to the previously reported method [22]. In the case of the studied systems, the solutions were first prepared in the same manner as in the solubility study. Then, the systems were diluted 1:1 with water, filtered through a 0.2 μm PTFE membrane filter, and further diluted 1:1 with DMSO. Next, the obtained solution was diluted 1:1 with donor solution for the GIT and BBB assays and placed in the donor compartment. The results were expressed as a concentration in the acceptor solution.

### 3.5. Biological Activities Studies

#### 3.5.1. Antioxidant Activity Assay

Briefly, 25.0 μL of studied solutions (prepared for concentration determination in a solubility study) was mixed with 175.0 μL of DPPH radical solution. The rest of the analysis was performed according to the outlined procedure [48].

#### 3.5.2. Determination of Butyrylcholinesterase (BuChE) Inhibition

The determination of BuChE inhibition was performed according to the previously reported method [48].

### 3.6. Statistical Analysis

Results are expressed as mean ± (standard deviation). Statistical tests were performed using a one-way analysis of variance (ANOVA), and statistical differences using Duncan’s tests with a significance threshold of *p* < 0.05 were determined. All statistical analyses were performed using Statistica 13.1 software (TIBCO Software Inc., Palo Alto, CA, USA).

## 4. Conclusions

To sum up, in this work, we successfully used hot-melt extrusion processing to produce amorphous systems of curcumin and piperine for a potential combined delivery. It is important to enhance the bioaccessibility of those compounds since it is a major limitation in their use as pro-health agents. The systems showed improved pharmaceutical features such as dissolution rate and solubility, which positively affected the passive permeability of plant-origin active compounds. Furthermore, the greater solubility provided by the systems boosted in vitro biological activity thanks to the increase in freely dispersed molecules in the aqueous solution that could act on biological targets. Obtaining amorphous systems of curcumin and piperine seems to be an effective approach to improving their pro-health potential.

## Figures and Tables

**Table 1:** The results of apparent solubility studies of amorphous systems are presented as achieved concentrations and folds of improvement with regard to curcumin and piperine.

System (Cur:Pip:VA 64)	Compound			
	CUR		PIP	
	Conc. (mg/mL)	Improv. (-fold)	Conc. (mg/mL)	Improv. (-fold)
Raw	0.0001 ± 0.0000 ^e^	-	0.006 ± 0.0001 ^e^	-
1:1:4	0.689 ± 0.002 ^d^	5514	0.153 ± 0.001 ^d^	23
1:1:8	0.910 ± 0.003 ^c^	7283	0.669 ± 0.028 ^c^	103
1:1:12	1.049 ± 0.009 ^b^	8396	0.826 ± 0.010 ^b^	138
1:1:16	1.187 ± 0.010 ^a^	9496	1.050 ± 0.016 ^a^	161

The statistically significant values are presented as ^a–e^, with “^a^” being the highest value (*p* < 0.05).

**Table 2:** The results of the in vitro permeability assay for curcumin and piperine. Amorphous systems were named as a mass ratio of individual components, Cur:Pip:VA 64 (Cur: curcumin, Pip: piperine, and VA 64: PVP VA 64).

System (Cur:Pip:VA 64)			Compound			
			CUR		PIP	
			Conc. (mg/mL)	Improv. (-fold)	Conc. (mg/mL)	Improv. (-fold)
Model	GIT	Raw	3.34 × 10^−6^ ± 1.94 × 10^−6, e^	-	5.86 × 10^−4^ ± 1.35 × 10^−4, e^	-
		1:1:4	1.09 × 10^−3^ ± 4.09 × 10^−4, d^	326	5.33 × 10^−3^ ± 1.76 × 10^−3, d^	9
		1:1:8	2.60 × 10^−2^ ± 4.57 × 10^−3, c^	7776	1.21 × 10^−1^ ± 8.88 × 10^−3, c^	207
		1:1:12	3.33 × 10^−2^ ± 2.87 × 10^−3, b^	9976	1.66 × 10^−1^ ± 1.09 × 10^−2, b^	284
		1:1:16	4.20 × 10^−2^ ± 2.21 × 10^−3, a^	12,578	2.01 × 10^−1^ ± 7.77 × 10^−3, a^	343
	BBB	Raw	1.86 × 10^−5^ ± 2.48 × 10^−6, e^	-	2.10 × 10^−3^ ± 8.86 × 10^−5, d^	-
		1:1:4	1.51 × 10^−2^ ± 1.86 × 10^−3, d^	815	2.06 × 10^−2^ ± 3.73 × 10^−3, c^	10
		1:1:8	4.12 × 10^−2^ ± 1.40 × 10^−3, c^	2218	2.99 × 10^−1^ ± 6.85 × 10^−3, b^	142
		1:1:12	4.45 × 10^−2^ ± 2.47 × 10^−3, b^	2436	3.25 × 10^−1^ ± 2.97 × 10^−2, a, b^	155
		1:1:16	5.70 × 10^−2^ ± 1.40 × 10^−3, a^	3069	3.45 × 10^−1^ ± 2.07 × 10^−2, a^	164

The statistically significant values are presented as ^a–e^, with “^a^” being the highest value (*p* < 0.05).

**Table 3:** The results of the amorphous systems’ improved inhibition activities against DPPH radicals and BChE.

System (Cur:Pip:VA 64)	Assay	
	DPPH	BChE
	% of Inhibition	% of Inhibition
Raw	10.69 ± 0.92 ^d^	1.24 ± 0.27 ^e^
1:1:4	48.16 ± 2.56 ^c^	42.87 ± 2.27 ^d^
1:1:8	87.87 ± 1.12 ^b^	77.46 ± 2.48 ^c^
1:1:12	96.85 ± 1.35 ^a^	92.35 ± 3.55 ^b^
1:1:16	96.97 ± 1.32 ^a^	98.52 ± 0.87 ^a^

The statistically significant values are presented as ^a–e^, with “^a^” being the highest value (*p* < 0.05).

## Data Availability

Data are available in a publicly accessible repository.

## Supplementary Materials

The following supporting information can be downloaded at: https://www.mdpi.com/article/10.3390/molecules28093848/s1, Figure S1: XRPD patterns of systems’ physical mixtures; Table S1: The most important peak positions of raw curcumin, piperine, and Kollidon VA64 with peak assignment; Figure S2: Physical stability studies XRPD patterns; Figure S3: Chromatograms of curcumin (a) at 420 nm and piperine (b) at 340 nm; Table S2: HPLC-DAD method validation parameters for curcumin and piperine determination;. References [49,50,51,52,53,54,55,56,57] are mentioned in the Supplementary Materials.

## Sample Availability

Samples of the raw curcumin, raw piperine, Kollidon VA64, and obtained amorphous systems are available from the authors.
