# Supplementary material for: Hot-Melt Extrusion as an Effective Technique for Obtaining an Amorphous System of Curcumin and Piperine with Improved Properties Essential for Their Better Biological Activities
Source: Molecules. 2023 May 1;28(9):3848. doi: 10.3390/molecules28093848 (PMC10180276; doi:10.3390/molecules28093848)
Supplement: Supplementary file 1 [file molecules-28-03848-s001.zip › molecules-2369763-supplementary.pdf]

molecules

Article

Hot-melt extrusion as an effective technique for obtaining an amorphous system of curcumin and piperine with improved properties essential for their better biological activities – Supplementary Materials

Kamil Wdowiak[1], Robert Pietrzak[2], Ewa Tykarska[3] and Judyta Cielecka-Piontek [1,*]

[1] Department of Pharmacognosy, Faculty of Pharmacy, Poznan University of Medical Sciences, Rokietnicka 3, 60-806 Poznan, Poland
[2] Faculty of Chemistry, Adam Mickiewicz University in Poznań, Uniwersytetu Poznańskiego 8, 61-614 Poznan, Poland
[3] Department of Chemical Technology of Drugs, Poznan University of Medical Sciences, Grunwaldzka 6, 60-780 Poznan, Poland
* Correspondence: jpiontek@ump.edu.pl

Copyright: © 2023 by the authors. Submitted for possible open access publication under the terms and conditions of the Creative Commons Attribution (CC BY) license (https://creativecommons.org/licenses/by/4.0/).

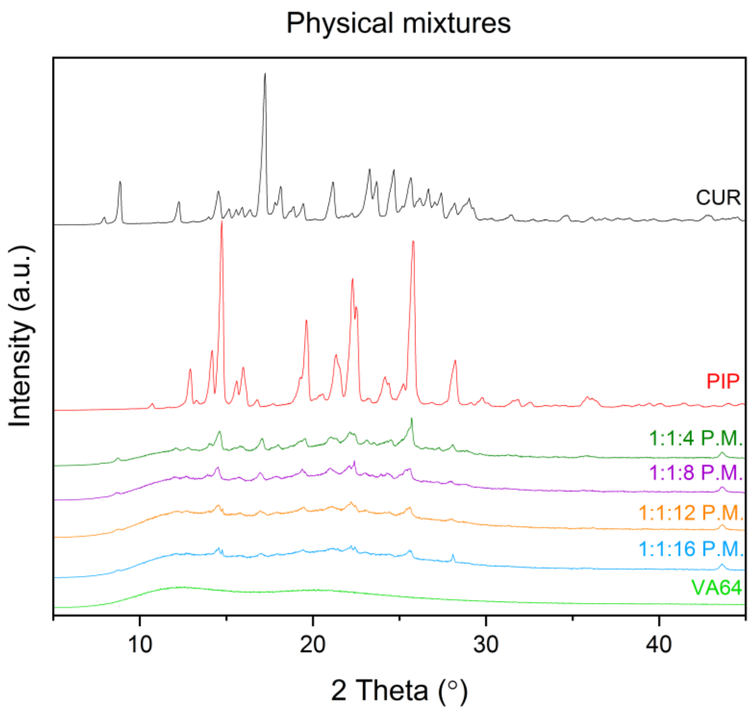

Figure S1. XRPD patterns of systems' physical mixtures. Physical mixtures of systems were named as a mass ratio of individual components, Cur:Pip:VA 64 (Cur: curcumin, Pip: piperine, and VA 64: PVP VA 64).

Table S1. The most important peak positions of raw curcumin, piperine, and Kollidon VA64 with peak assignment. The assignment of curcumin was performed based on [49, 50, 51], piperine based on [52, 53, 54, 55], and Kollidon VA64 based on [56, 57].

**Curcumin**	
**Wavelength of peak (cm^{-1})**	**Peak assignment**
3502	Phenolic -OH stretching
1602	C=C stretching of aromatic rings
1627	C=C and C=O stretching vibrations of inter-ring chain
1601	C=C stretching of aromatic rings
1507	C=O stretching and bending of C=O and C=C
1426	C-H plane bending
1275	CH of C=CH bending and CCH stretching of the aromatic ring in the enolic side of the molecule
1152	C-O vibrations and aromatic C-H bending
**Piperine**	
2942	C-H stretching
1634	N-H bending
1582	N-C=O stretching in carbonyl amide
1512	C-NH bending and C=C stretching of aromatic ring
1433	$C=CH_2$ CH_2 deformation and bending
1368	C-H bending
1310	C-N stretching
1249	C-O stretching in methylenedioxy group

927	C-O vibration in methylenedioxyphenyl
**Kollidon VA64**	
1730	C=O stretching in vinyl acetate group
1668	C=O stretching in pyrrolidone ring
1290	C-N stretching

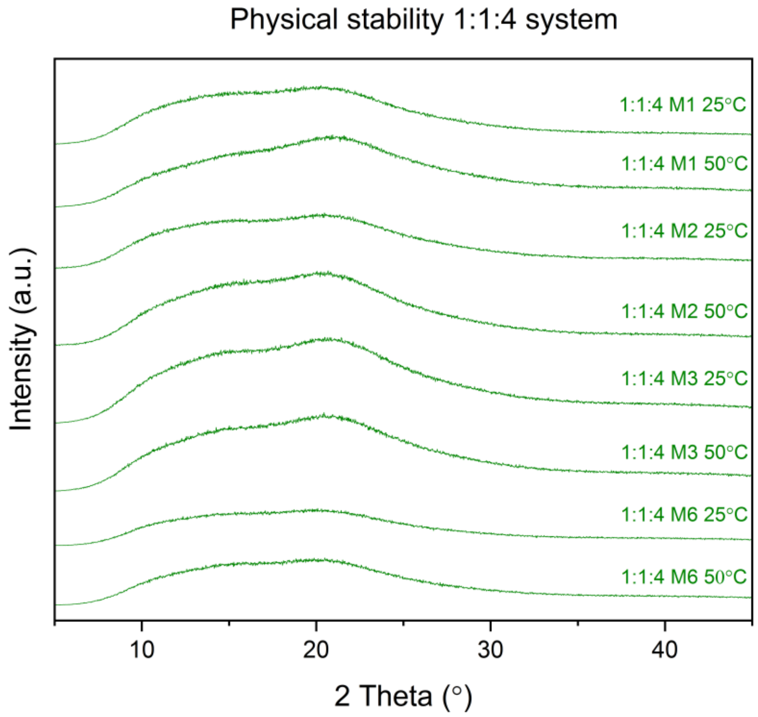

(a)

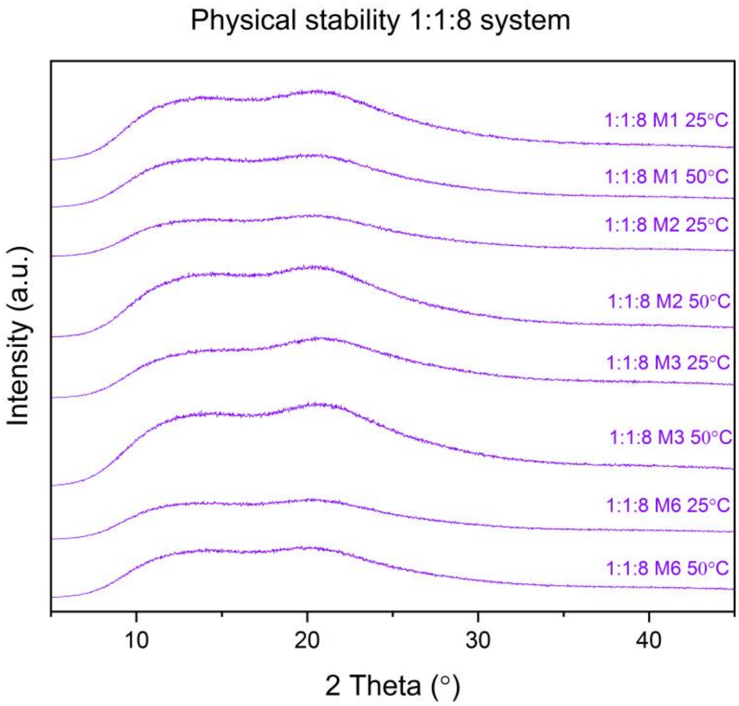
Physical stability 1:1:8 system
Intensity (a.u.)
2 Theta (°)
10
20
30
40
1:1:8 M1 25°C
1:1:8 M1 50°C
1:1:8 M2 25°C
1:1:8 M2 50°C
1:1:8 M3 25°C
1:1:8 M3 50°C
1:1:8 M6 25°C
1:1:8 M6 50°C

(b)

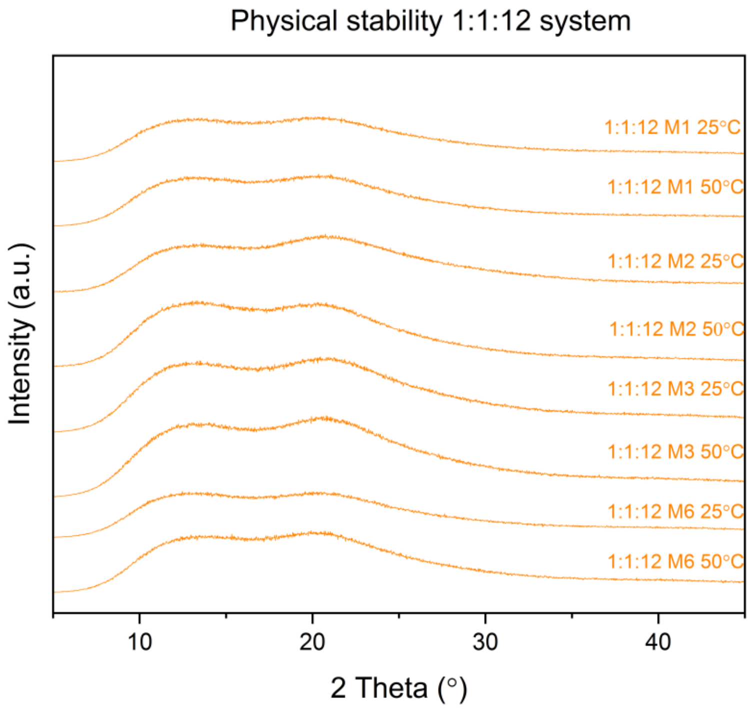
Physical stability 1:1:12 system
Intensity (a.u.)
2 Theta (°)
10
20
30
40
1:1:12 M1 25°C
1:1:12 M1 50°C
1:1:12 M2 25°C
1:1:12 M2 50°C
1:1:12 M3 25°C
1:1:12 M3 50°C
1:1:12 M6 25°C
1:1:12 M6 50°C

(c)

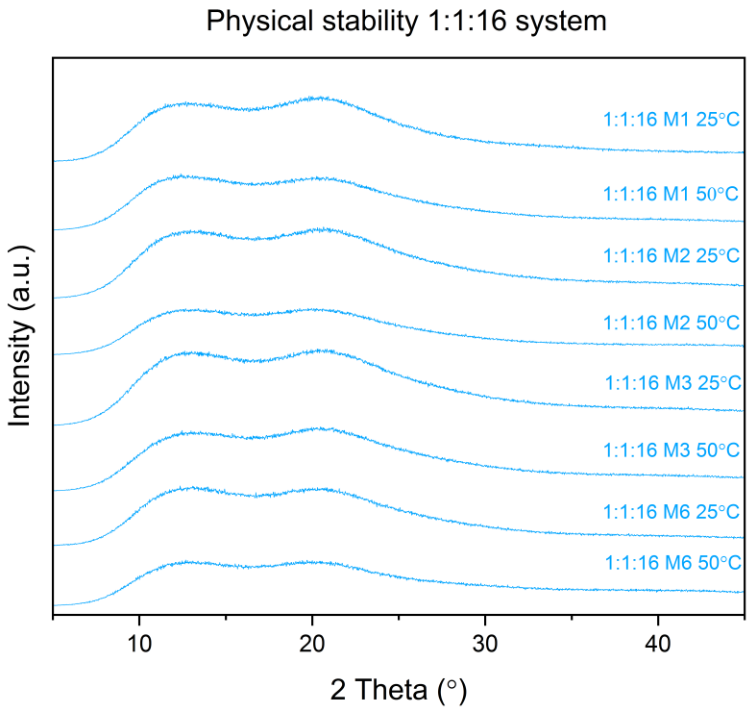

(d)

Figure S2. Physical stability XRPD patterns of amorphous systems. Amorphous systems were named as a mass ratio of individual components, Cur:Pip:VA 64 (Cur: curcumin, Pip: piperine, and VA 64: PVP VA 64). “M” = month; (a) physical stability of 1:1:4 amorphous system, (b) physical stability of 1:1:8 amorphous system, (c) physical stability of 1:1:12 amorphous system, (d) physical stability of 1:1:16 amorphous system.

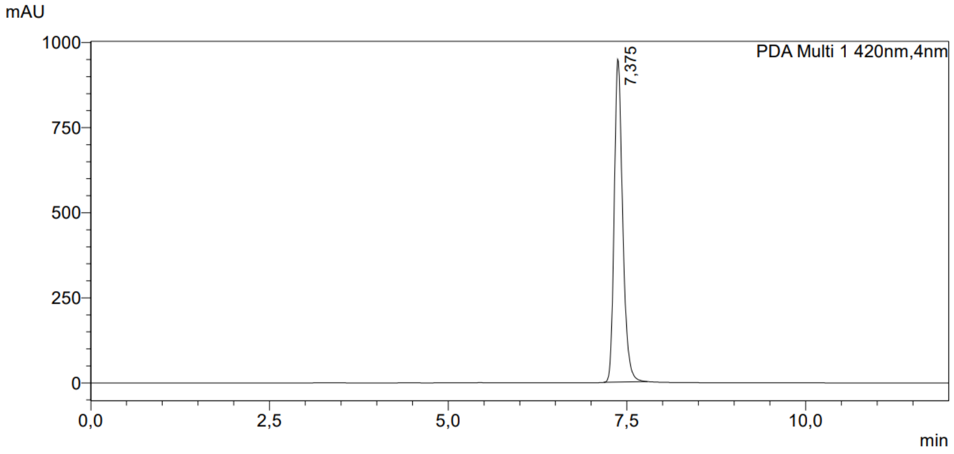

(**a**)

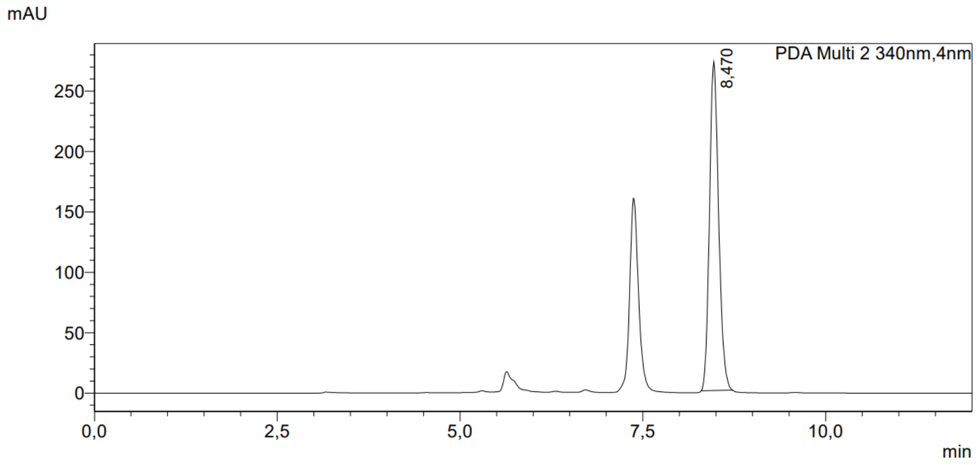

(**b**)

Figure S3. Chromatograms of curcumin (a) at 420 nm and piperine (b) at 340 nm.

Chromatographic conditions:

- Stationary phase - Dr. Maisch ReproSil-Pur Basic-C18 100 Å column, 5 µm particle size, 250 × 4.60 mm
- Mobile phase - methanol/0.1% acetic acid (80:20 v/v)
- Column temperature - 30 °C
- Flow rate - 1.0 mL/min

Table S2. HPLC-DAD method validation parameters for curcumin and piperine determination.

**Curcumin**	
Parameter	Curcumin dissolved in 50% DMSO; Injection volume 10 µl
Linearity range (mg/mL)	0.00005 – 0.2
Correlation coefficient (r)	0.9999
$a \pm S_a$	177526161 ± 1591328
$b \pm S_b$	insignificant (α=0.05)
LOD (mg/mL)	0.0026
LOQ (mg/mL)	0.0079
Retention Time	7.375
**Piperine**	
Parameter	Piperine dissolved in 50% DMSO; Injection volume 10 µl
Linearity range (mg/mL)	0.00005 – 0.2
Correlation coefficient (r)	1.0000
$a \pm S_a$	158068711 ± 276319
$b \pm S_b$	insignificant (α=0.05)
LOD (mg/mL)	0.0005
LOQ (mg/mL)	0.0015
Retention Time (min)	8.470

Disclaimer/Publisher's Note: The statements, opinions and data contained in all publications are solely those of the individual author(s) and contributor(s) and not of MDPI and/or the editor(s). MDPI and/or the editor(s) disclaim responsibility for any injury to people or property resulting from any ideas, methods, instructions or products referred to in the content.
